# The Summer Vacation

**Published:** 1888-09

**Authors:** 


### HALL'S JOURNAL OF HEALTH

TRUTH DEMANDS NO SACRIFICE; ERROR CAN MAKE NONE.

VOL. 35. SEPTEMBER, 1888. No. 9.

### THE SUMMER VACATION.

The outing season, as the summer vacation is termed, is nearly over. To the opulent and unemployed, who have all seasons for their own, it signifies a gaily time at the resorts of fashion, where coy young maidens are first "brought out," and fortunes played like a foot-ball amid gay youths with family backing, and scrambling adventurers who offer an empty title in trade to prop a falling house.

But to the weary and over-worked denizons of our crowded cities, whose necessities are masters of their time, the few days of summer vacation are looked forward to with an eagerness akin to that of the prisoner to the period of his enlargement. And blessed days they are, too, when the drudgery of everyday life is laid aside, though ever so briefly, for free, health-restoring rambles over the verdure clad hills and dales of our beautiful rural surroundings.

> "There is a pleasure in the pathless woods,
> There is a rapture on the lonely shore,
> There is society where none intrudes,
> By the deep sea, and music in its roar:"

Ere another month all of this deserving class will have returned to resume their wonted duties in the stores, workshops and counting houses of our various industrial centres, there to give to a long period of almost unremitting toil, the renewed energies with which a few days of sunshine have strengthened their lives, yet, who shall say that these alternations of labor and diversion that so unequally divide their days, are not far preferable to a life given over to luxury and ease? There is no needless waste of time; no corroding dissipation of either body or mind, which so often overtakes the idle and aimless on their downward road. The very duties which necessity enjoins oftentimes prove a saving grace. Every one ought to have some suitable employment, if only as a sanitary measure. In lack of it the body becomes inert and inelastic, like an unworked and over-fed animal, and the mind which depends upon it for healthfulness, is sure to sink to the level of its inertia. Eternal motion is the order of the universe. Without this there comes stagnation, and stagnation means poison and death.

The humblest laborer who is faithful to his trust leaves the world something the better for his being of it ; adds something to the commonweal.

"Love labor ; for if thou dost not want it for food, thou mayest for physic," was an axiom of no less a personage than the founder of Pennsylvania. Nothing is gained to mankind in knowledge or experience except by labor, and he who honors it not is unworthy to partake of its bounty. In the words of Carlyle :

> "Labor, wide as the earth, has its summit in heaven,
> None can begin at the summit."

